# Pediatric testicular microlithiasis: a review of epidemiology, risk associations and management

**DOI:** 10.3389/fped.2025.1553825

**Published:** 2026-01-13

**Authors:** Hayet Zitouni, Rim Dghaies, Nahla Kechiche, Riadh Mhiri

**Affiliations:** 1 Department of Pediatric Surgery, Hedi Chaker Hospital, Sfax, Tunisia; 2 University of Sfax, Sfax, Tunisia; 3 Department of Pediatric Surgery, Fattouma Bourguiba University Hospital, Monastir, Tunisia

**Keywords:** prevalence, children, testicular microlithiasis, histology, management

## Abstract

Testicular microlithiasis is defined by the presence of five or more randomly distributed hyperechogenic microliths, each measuring less than 3 mm in diameter, observed in a single ultrasound scan. Histologically, testicular microlithiasis is characterized by multiple laminated calcifications within seminiferous tubules. The underlying cause of these calcifications is not yet fully understood. The exact prevalence of testicular microlithiasis in children remains uncertain and continues to be a topic of debate. These calcifications can occur either independently or in association with various benign conditions, malignant pathologies, or specific congenital disorders. Whether these associations are causal or coincidental remains unclear. The aim of this study is to review the prevalence of TM in children, with emphasis on its histological characteristics, underlying mechanisms, and contributing factors, and to investigate its association with testicular malignancy in order to propose a pragmatic follow-up approach based on current guideline recommendations.

## 1 Introduction

Testicular microlithiasis (TM) in children is a rare clinical condition characterized by multiple small intratesticular calcifications detected through radiographic or, more commonly, ultrasound imaging (1). Histologically, TM is defined by the presence of multiple laminated calcifications within the seminiferous tubules (2). The underlying mechanisms are not fully understood. Proposed explanations include testicular tubular degeneration, impaired Sertoli cell phagocytosis with accumulation of epithelial cell debris, subsequent inflammatory and immune responses, or the presence of a genetic mutation (2, 3).

The first documented case of TM was reported in 1970 in a 4-year-old boy, identified incidentally during a pelvic X-ray (4). Although TM can occur at any age, it is more frequently reported in childhood (3), with an estimated prevalence of 4.2% (5, 6). However, its true incidence remains uncertain.

TM is more often observed in association with specific congenital disorders, such as undescended testes (UDT), Down syndrome, Klinefelter syndrome, McCune–Albright syndrome, and Peutz–Jeghers syndrome (7). It may also be linked to other testicular conditions, including testicular torsion, torsion of the appendix testis, primary testicular neoplasms, intratubular germ cell neoplasia, benign cystic teratoma, varicocele, and even in otherwise normal testes. Whether these associations are causal or coincidental remains unclear (8).

This study is a narrative review based on published literature. Its aim is to synthesize current evidence on the prevalence of TM in children, with particular attention to its histological features, contributing factors and potential association with testicular malignancy. We also propose a pragmatic follow-up approach based on current guideline recommendations.

## 2 Definition

Testicular microlithiasis is a rare, usually asymptomatic condition, characterized by diffuse calcifications within the seminiferous tubules. Historically, diagnosis was made through testicular biopsy, but it is now most commonly established using high-frequency testicular ultrasound (9).

### 2.1 Histological definition

Testicular calcifications are heterogeneous, and at least three histological types have been described:

- *hematoxylin bodies,* consisting of amorphous calcified material;
- *ossification,* consisting of bone tissue; and
- *laminated or psammomatous calcifications* (2).

By definition, TM requires the demonstration of multiple laminated calcifications within the seminiferous tubules (2). Histologically, microliths—also termed microcalcospherites—are easily recognized. They appear as rounded hematoxyphilic bodies composed of stratified, concentric blue rings. Their size may be smaller than, equal to, or larger than adjacent seminiferous tubules. Narrow, pale, hyaline peripheral rings, bordered by connective tissue, may also be present (10).

The histological definition of testicular microlithiasis requires clarification. Isolated laminated concretions alone are insufficient for a definitive diagnosis, as they are relatively common in otherwise normal testes. For example, laminated calcifications have been reported in approximately 50% of cryptorchid testes, 40% of testes with germ cell tumors (GCT), and 4% of normal testes, as reported by Geode et al. (6). Furthermore, hematoxylin bodies, which occur only in association with an existing tumor or its scar, should be clearly distinguished from testicular microlithiasis (6).

### 2.2 Radiological definition

By definition, TM is diagnosed when five or more randomly distributed hyperechogenic microliths, each measuring less than 3 mm in diameter, are observed in a single ultrasound scan. These calcifications may be diffusely or focally distributed and can occur unilaterally or bilaterally (11).

The typical sonographic appearance consists of multiple tiny echogenic foci, most often distributed bilaterally, diffusely, and symmetrically within the testicular parenchyma (8).

Goede et al. (6) further classified TM as follows:

- Classic TM (CTM): ≥5 echogenic foci.
- Limited TM (LTM): <5 echogenic foci.

In addition, TM may be categorized by its distribution within the parenchyma as diffuse or segmental.

## 3 Etiology

The etiology of TM remains unclear, and several mechanisms have been proposed (12). One hypothesis suggests that the impaired phagocytic function of Sertoli cells leads to the accumulation of epithelial debris within the seminiferous tubules (2). This may initiate inflammatory and immune responses and increase basal membrane permeability, promoting the formation of deposits in both the tubular lumen and the interstitium (2, 10).

Another theory proposes that the seminiferous epithelium undergoes a degenerative process of unknown origin, producing cellular debris that migrates into the tubular lumen and acts as a calcification nidus (4, 13).

These theories could explain the frequency of TM in children, as more rapid cellular turnover in children leads to increased luminal debris.

The hypothesis that microliths originate from ectopic oocytes in dysgenetic testes is unsupported, as similar calcifications are also found in otherwise normal testes in both children and adults with normal testicular function and fertility (3, 14).

Given the current uncertainty, further studies are needed, as a clearer understanding of the pathogenesis of TM may help elucidate its potential relationship with testicular tumors.

## 4 Epidemiology and clinical correlations

### 4.1 Historical background and prevalence

Testicular microlithiasis (TM) was long regarded as a rare condition (10). The first case was reported by Priebe and Garret in 1970, in a healthy 4-year-old boy (4). However, with the increasing use of high-resolution inguinoscrotal ultrasound in recent years, TM has been recognized as more common than previously thought (11).

### 4.2 Symptomatic and asymptomatic children

The exact prevalence of TM in children remains uncertain and continues to be debated. In adults, reported prevalence rates range from 0.6% to 9%, depending on study population and methodology, with a generally accepted estimate of around 2% among symptomatic individuals undergoing ultrasound examinations (15).

Patients are considered symptomatic if ultrasound is performed due to scrotal complaints, and asymptomatic if no such complaints are present (16).

In pediatric populations, TM has been identified in asymptomatic children with a prevalence of 4.2% in several studies (5, 6). In contrast, its prevalence among symptomatic boys is lower, at approximately 2.0% (17, 18). This difference may be explained by the more recent recognition of TM in asymptomatic populations, advances in diagnostic tools, and the retrospective design and small sample sizes of earlier studies (16). Furthermore, several studies suggest that TM prevalence increases with age, even in otherwise healthy children (6, 7), which may also account for these discrepancies (9).

### 4.3 Associated benign testicular pathologies

TM can occur in isolation or in association with a variety of benign testicular pathologies, including testicular torsion, appendix testis torsion, undescended testes (UDT), hydrocele, testicular atrophy, varicocele, and infections affecting the testes and epididymis (8). The reported prevalence of TM in patients with UDT ranges from 2.8% to 12% (7, 16). In the study by Dutra et al. (11), up to 10% of children with cryptorchidism were found to have TM, which is approximately twice the incidence observed in boys with normally descended testes.

Conversely, patients with inguinal hernia demonstrated a markedly reduced risk of TM. With regard to testicular hypotrophy, only a single case was reported in association with TM, suggesting that the calculated relative risk (79.11) is likely unreliable.

In a study by Goede et al. (16), the overall prevalence of TM in UDT patients was 2.8%, with 2.2% classified as classic TM (CTM) and 0.6% as limited TM (LTM). No significant difference was observed between congenital and acquired (ascending) UDT. These rates were slightly lower than the 4.2% prevalence reported in asymptomatic boys (16).

Although congenital UDT is generally treated early, the prevalence of TM in patients undergoing surgery at the optimal age remains uncertain (7, 16). Taskinen et al. (19) reported abnormalities in testicular tissue in 17% of UDT cases treated in childhood, suggesting that testicular damage in UDT may result not only from the underlying condition but also from the surgical intervention itself.

### 4.4 Associated syndromes

TM has also been observed in association with multiple genetic conditions, including Down syndrome (20), Klinefelter syndrome (21), some forms of intersexuality, alveolar microlithiasis, and disorders of the sympathetic nervous system. Whether these associations are causal or coincidental remains unclear (8). Goede et al. (20) reported a 22.8% prevalence of testicular microlithiasis (TM) in boys with Down syndrome, while Cebeci et al. (22) found an even higher rate of 36%. Both rates were markedly greater than the 4.2% observed in boys without Down syndrome. Similarly, a high prevalence has been reported in boys with McCune–Albright syndrome, reaching 62% in the study by Wasniewska et al. (23).

### 4.5 Association with testicular malignancies

In adults, TM has historically been associated with an increased risk of GCT (24, 25). However, current evidence indicates that isolated TM, in the absence of additional risk factors—such as prior GCT, a family history of GCT in a first-degree relative, undescended testis or orchidopexy, or testicular atrophy—represents a benign finding that does not warrant routine surveillance (17, 26–29). In a longitudinal study (mean follow-up: 18 months, range up to 165 months), only 2 out of 442 adults with TM developed testicular cancer (27). The two incidents occurred in those with additional risk factors such as testicular hypotrophy.

In children, the association between TM and malignancy remains controversial. There is a lack of long-term studies following children with TM to determine their risk of developing testicular tumors. In a study by Kocaoglu et al. (30), nine children with TM (aged 3–16 years) were followed for 6 months to 6 years, with no tumors observed. Furness et al. (31) conducted a multicenter study of 26 children (aged 0.5–21 years) with TM, with a mean follow-up of 27.6 months, and reported no cases of tumor development. Similarly, Leenen et al. (32) followed six of 16 patients with TM for up to 6 years without detecting tumors. These follow-up periods may have been too short, since testicular tumors typically develop between the ages of 20 and 40 years (33).

Most pediatric case series have not demonstrated a clear link between TM and testicular cancer, with only a few isolated cases reported. Evidence suggests that in some instances, TM may coexist with, rather than directly cause, tumors (34, 35). In a systematic review and meta-analysis published in 2021 by ‘t Hoen Lisette et al. (36), including 595 children with TM, only one patient developed testicular malignancy during follow-up.

Thus, distinguishing true tumor development from incidental coexistence of TM and testicular tumors remains essential (Table 1) (10, 15, 37–44).

**Table 1:** Association with testicular malignancies in children.

Author, year	Age at TM diagnosis	Development/coexistence of tumor	Nature of tumor
McEniff et al. (37), 1995	17 years old	Tumor development	Testicular germ cell tumor
Arrigo et al. (38), 2006	6 years old	Tumor development	Testicular germ cell tumor
Slaughenhoupt et al. (39), 2009	11 years old	Tumor development	Metastatic mixed germ cell tumor
Toh et al. (40), 2000	16 years old	Concomitant diagnosis of TM and tumor	Metastatic mixed germ cell tumor
Drut et al. (10), 2002	16 years old	Concomitant diagnosis of TM and tumor	Testicular germ cell tumor
Drut (41), 2003	2 years old	Concomitant diagnosis of TM and tumor	Yolk sac tumor
Nistal et al. (42), 2004	4 years old	Concomitant diagnosis of TM and tumor	Rete testis tumor
Meyer et al. (43), 2010	16 years old	Concomitant diagnosis of TM and tumor	Retroperitoneal extra gonadal sinus tumor
Deganello et al. (15), 2012	11 years old	Concomitant diagnosis of TM and tumor	teratoma

### 4.6 Future fertility risk

No studies have specifically examined the relationship between TM diagnosed in prepubertal boys and the risk of infertility in adulthood (36), particularly given that most research on TM in children has involved short follow-up periods ending at puberty.

### 4.7 Resolution possibility

It has been suggested that TM detected at an early age may not necessarily be permanent and could regress over time, potentially explaining the variation in reported prevalence rates (15). In a study by ‘t Hoen Lisette et al. (36), among 595 patients with testicular microlithiasis, regression or complete resolution of TM was observed in 33 testes during follow-up.

## 5 Management of testicular microlithiasis and international guidelines

The exact relationship between TM and both benign and malignant disorders remains uncertain, complicating follow-up and management. In adults, surveillance practices have varied widely, including self-examinations, annual or biannual ultrasound scans, tumor marker screening, and even biopsy (36). However, current studies (36, 44) suggest that boys should only begin self-examination at around age 15, with no additional routine surveillance recommended. Goede et al. (44) further advise ultrasound follow-up only in cases of pain, testicular enlargement, or the presence of risk factors.

Both European and American societies agree that isolated TM requires no routine follow-up. However, their recommendations diverge in cases where additional risk factors are present.

### 5.1 European guidelines

The European Society of Urogenital Radiology (ESUR) and the European Association of Urology (EAU) advocate a proactive approach in adults with TM and risk factors. They recommend annual ultrasound until midlife, in addition to education on testicular self-examination (28, 45). For children and adolescents, the EAU advises against routine imaging or contralateral biopsy but emphasizes self-examination during and after puberty if risk factors exist (45, 46).

### 5.2 American guidelines

The American Urological Association (AUA) and the American College of Radiology (ACR) adopt a more conservative strategy. Even in the presence of risk factors, they do not recommend scheduled ultrasound surveillance, instead focusing on education, clinical awareness, and selective imaging when symptoms or palpable abnormalities occur (17, 29, 47). This difference underscores the ongoing uncertainty regarding optimal surveillance and suggests that follow-up should be individualized, balancing patient risk profile, psychological impact, and healthcare resource use (Table 2)

**Table 2:** Follow-up guidelines summary of TM.

Group	Europe (ESUR/EAU)	United States (AUA/ACR)
Adults (≥18 years)	Isolated TM (no risk factors): No routine follow-up; teach self-examination. TM + risk factors: Annual scrotal ultrasound until approximately 55 years; monthly self-exam; urgent referral if palpable mass (45, 46).	Isolated TM: No increased malignancy risk. No follow-up or further evaluation needed. TM + risk factors: No routine ultrasound or biopsy recommended; emphasis on education and self-examination. Ultrasonography may be considered selectively (17, 29, 47).
Children/adolescents	Isolated TM: No routine ultrasound; no contralateral biopsy in prepubertal boys. TM + risk factors: Inform family; encourage self-examination during/after puberty; no routine imaging (45, 46).	Isolated TM: No routine follow-up (Equivalent approach). TM + risk factors: Educate and encourage testicular self-examination after puberty; no formal surveillance imaging guidelines (17, 29, 47). (No dedicated pediatric guideline exists, but consensus follows adult-focused recommendations.)

## 6 Clinical practice considerations

According to this review, the prevalence of microlithiasis is notably lower in symptomatic patients and those with cryptorchidism compared with asymptomatic children. This raises questions about the etiopathogenesis and contributing factors of TM. It also raises the possibility that TM may regress over time, which, if confirmed, could support a strategy of clinical follow-up alone without further investigations.

The perception of TM has changed considerably. Once regarded as a marker of increased germ cell tumor (GCT) risk (18, 19), isolated TM is now recognized as a benign finding with no inherent malignant potential, requiring no systematic surveillance in either adults or children (21–24).

Regarding these findings, a pragmatic approach may be to apply the European model of annual ultrasound in high-risk adults while adopting the American strategy of reassurance and self-examination for isolated TM or low-risk cases. For children, self-examination is recommended only in the presence of risk factors. Importantly, prepubertal boys and their parents should be referred to an adult urologist to discuss the need for long-term follow-up into midlife.

## 7 Limitations of current evidence

While this review provides an overview of the current evidence on TM in children, several methodological limitations must be acknowledged. Most pediatric studies are retrospective, involve small cohorts, and have short follow-up periods. These limitations prevent reliable assessment of how TM detected in childhood evolves into adulthood and raise the question of whether TM identified in children represents the same entity as TM in adults. Consequently, there is a clear need for prospective studies with larger cohorts and long-term follow-up to better define the clinical significance of TM in children and clarify potential associations with testicular malignancy and fertility outcomes.

## 8 Conclusion

Although testicular microlithiasis has been recognized for some time, it remains relatively rare in clinical practice. The exact cause of this condition is still unknown, and its prevalence is uncertain due to its asymptomatic nature, which limits detection in the general population. In the absence of risk factors, isolated TM in children seems to be benign and does not necessitate long-term monitoring. However, prospective multicenter studies are required to delineate long-term fertility and cancer outcomes.
